# Response of Carrot (*Daucus carota* L.) to Multi-Contaminated Soil from Historic Mining and Smelting Activities

**DOI:** 10.3390/ijms242417345

**Published:** 2023-12-11

**Authors:** Milan Novák, Veronika Zemanová, Marie Lhotská, Milan Pavlík, Aleš Klement, František Hnilička, Daniela Pavlíková

**Affiliations:** 1 Department of Agroenvironmental Chemistry and Plant Nutrition, Faculty of Agrobiology, Food and Natural Resources, Czech University of Life Sciences Prague, Kamýcká 129, 16500 Prague, Czech Republic; milannovak@fld.czu.cz (M.N.);; 2 Department of Botany and Plant Physiology, Faculty of Agrobiology, Food and Natural Resources, Czech University of Life Sciences Prague, Kamýcká 129, 16500 Prague, Czech Republic; lhotskamarie@af.czu.cz (M.L.); hnilicka@af.czu.cz (F.H.); 3 Department of Soil Science and Soil Protection, Faculty of Agrobiology, Food and Natural Resources, Czech University of Life Sciences Prague, Kamýcká 129, 16500 Prague, Czech Republic; klement@af.czu.cz

**Keywords:** cadmium, free amino acids, lead, malondialdehyde, photosynthesis, root vegetable, soil enzymes, zinc

## Abstract

A pot experiment was undertaken to investigate the effect of Cd, Pb and Zn multi-contamination on the physiological and metabolic response of carrot (*Daucus carota* L.) after 98 days of growth under greenhouse conditions. Multi-contamination had a higher negative influence on leaves (the highest Cd and Zn accumulation) compared to the roots, which showed no visible change in terms of anatomy and morphology. The results showed the following: (i) significantly higher accumulation of Cd, Zn, and Pb in the multi-contaminated variant (Multi) compared to the control; (ii) significant metabolic responses—an increase in the malondialdehyde content of the Multi variant compared to the control in the roots (by 20%), as well as in the leaves (by 53%); carotenoid content in roots decreased by 31% in the Multi variant compared with the control; and changes in free amino acids, especially those related to plant stress responses. The determination of hydroxyproline and sarcosine may reflect the higher sensitivity of carrot leaves to multi-contamination in comparison to roots. A similar trend was observed for the content of free methionine (significant increase of 31% only in leaves); (iii) physiological responses (significant decreases in biomass, changes in gas-exchange parameters and chlorophyll a); and (iv) significant changes in enzymatic activities (chitinase, alanine aminopeptidase, acid phosphatase) in the root zone.

## 1. Introduction

Toxic elements (TEs) are among the most severe pollutants that contaminate the environment. The exposure of the environment to toxic element contamination is increasing with the increase in urbanisation and industrialisation. Both geological sources and anthropogenic activities such as thermal power plants, mining and smelting activities, and waste processing contribute to this pollution [1]. Their severity is mainly due to their high toxicity and biological non-degradability [2,3]. Some TEs, such as zinc (Zn), are necessary for life because they are involved in many metabolic mechanisms, but in high concentrations, they can be toxic to organisms; however, cadmium (Cd) and lead (Pb) are toxic even in lower doses [4].

A significant issue is the contamination of TE soils, which leads to a negative impact on soil properties and a limitation of production and environmental functions [5]. Soil enzymes perform an important function in soil biochemical activities. For example, chitinase (CHIA) plays a key role in the degradation of chitin [6], and alanine aminopeptidase (AAP) catalyses the proteolysis of alanine and other hydrophobic amino acids (AAs) from the N-terminus of the polypeptide chain, releasing free AAs [7]. Due to the important role of enzymes in nutrient cycling, they are strong indicators of soil productivity and quality [8]. Acid phosphatase (PACID), which plays a key role in soil phosphorus cycling, is highly sensitive to TEs, making it a remarkable indicator of soil contamination [9,10].

The uptake of TEs by plant roots is a complex process managed by many factors that influence each other: plant species, genotype, availability, mobility and oxidation state of TEs in soil and soil properties. The cation-exchange capacity, pH, organic matter content, redox potential and interactions between elements through competitive and/or synergic mechanisms can limit the availability of TEs in soils [4,11,12]. TEs are passed to the roots, along with nutrients, through the root zone. It is an area at the root–soil interface with a high population of microorganisms, which the plant controls by releasing exudates and in which a plethora of interacting physical, chemical and biological processes occur [13]. Plants receive TEs dissolved in the soil solution either in ionic or chelated and complexed form and then either stored mainly by compartmentalisation and vacuolar sequestration or subsequently transported to aboveground parts mainly via the xylem by apoplastic (passive) and symplastic (active) transport [14]. The translocation of different TEs from roots to aboveground differs significantly. Compared to Cd and Zn, Pb has lower mobility, and most of it remains in the roots [15,16].

When plants interact with TEs, there is a conflict where, on the one hand, the toxicity of the TEs proves to have negative effects on the plant, and on the other hand, plant mechanisms deal with resistance to toxic effects and detoxification of the TEs [17].

Plants have complex defence mechanisms consisting of uptake/efflux, transport/sequestration and chelation [18]. Amino acids play an essential role in the development of plants in TE-contaminated environments [19]. These organic compounds are fundamentally involved in the chelation of metal ions. In this way, they reduce the concentrations of free metal ions and reduce the phytotoxicity caused by TEs [20]. In cases involving exposure to moderate TE concentrations, defence mechanisms can save the plant and allow its further growth and development. However, these strategies alone are not sufficient to mitigate the effects of long-term exposure to a high TE content, which causes TE toxicity in cells [21]. In this case, the amount of reactive oxygen species (ROS) may increase. ROS overproduction results in the modification of cellular AAs, membrane lipids, DNA and proteins. These reactions between ROS and cellular components cause mitochondrial dysfunction, DNA damage, cell membrane damage and, ultimately, cell death [22,23]. Plants grown in soils with high Cd, Pb and Zn content have visible damage symptoms, such as yield loss, growth inhibition, chlorosis, browning of root tips and death [24,25]. Both Cd and Zn have many physical and chemical similarities, and therefore their availability for plants is affected by their interaction, which is usually synergistic in nature [26]. According to Yang et al. [27], high Zn content can limit Cd uptake by vegetables.

Several studies documented that Cd toxicity causes the overproduction of oxidative markers like ROS, free radicals, and lipid peroxidation, which induces oxidative stress and has a negative effect on plant growth and yield. This element causes a significant reduction in carbon metabolism. This reduction leads to inhibition of the photosynthetic process including disruption in the chloroplast ultrastructure, inhibition of Calvin cycle enzymes, chlorophyll biosynthesis, impairment in electron transport, reduction in CO_2,_ assimilation due to stomatal closure and alteration of water, and nutrient equilibrium in plants [28,29].

In contrast to the previously mentioned element, Zn is an important plant micronutrient since it is involved in many key cellular functions such as metabolic and physiological processes, enzyme activation and ion homeostasis [30]. Zinc toxicity provokes deficiency of other nutrients owing to similar ionic radii and interference with their uptake and movement inside plants. Zinc excess causes a reduction in photosynthesis, transpiration and several other essential physiological processes [31,32]. Phytotoxic Zn concentrations enhance lipoxygenase activity, stimulate peroxidation of lipids (malondialdehyde-MDA) and influence membrane integrity and permeability [33].

Plants growing in soil containing considerable amounts of Pb accumulate higher levels of this element in roots, and only a small fraction is transported to aboveground plant parts [34,35,36]. Dogan et al. [37] showed that Pb accumulates primarily in root cells because of the blockage by Casparian strips. Results of Ghani et al. [35] showed that Pb stress retarded the plant growth and reduced chlorophyll content in the leaves of carrot cultivars. The decrease in the chlorophyll content was possibly affected by Pb binding to the SH group of enzymes of chlorophyll biosynthesis along with lipid peroxidation degradation. According to Khan et al. [38] and Shahid et al. [23], Pb accumulation in plants causes a decrease in photosynthetic rate, stopping the synthesis of chlorophyll, affecting the Calvin cycle, and causing a deficiency in CO_2_ that leads to the closure of stomata. Pb affects plant transpiration by reducing stomatal conductance, and thus the transpiration rate [39].

Vegetables are sensitive to TEs and easily accumulate TEs in their roots, stems and leaves [3]. One of the most important vegetables is the carrot (*Daucus carrota* L.), which has been cultivated for more than 1000 years and is one of the most popular vegetables worldwide. Currently, they are cultivated mainly in Europe and Asia [40,41]. Studies have demonstrated a hazardous TE content in carrot roots growing in contaminated soils [42,43]. In this study, the difference in carrot development in soil not contaminated or contaminated with a mixture of TEs was observed under semi-controlled conditions in a greenhouse. Carrots were exposed to TEs from seed sowing to consumption maturity. This study focused on investigating the changes in the physiological and metabolic response of carrots growing on Cd, Zn and Pb multi-contaminated soil in contrast to the control variant. The aim of this study was to determine changes in terms of anatomy and morphology of roots, changes in gas-exchange parameters and chlorophyll in leaves, and to show response to oxidative stress (demonstrated by changes in free amino acids, malondialdehyde and carotenoid content in roots).

## 2. Results

### 2.1. Cd, Pb and Zn Content in Carrots

As shown in Table 1, the accumulation of Cd, Pb and Zn was significantly higher in the multi-contaminated variant (Multi) compared to the control. Compared with the control, the average Cd content in the roots, periderm and leaves of the Multi variant was 24-, 42- and 46-fold higher, respectively. Additionally, the average Zn content of the roots, periderm and leaves was 2.4-, 1.4- and 3.8-fold higher, respectively. The highest change was determined in the case of Pb, whose content was under the detection limit in the control, and reached from 16.3 to 68.8 mg/kg DW in the Multi variant. This element predominantly accumulates in carrot roots. In contrast, Cd and Zn were translocated to the leaves, as was calculated by the translocation factor, which reached 1.8 to 2.9 for Cd in the control and the Multi variant, respectively, and 1.6 to 2.6 for Zn in the control and the Multi variant, respectively. In the Multi variant, a significant increase in Cd and Zn content of 188% and 155%, respectively, was confirmed in the leaves compared to the roots. The Pb content was significantly reduced by 59% in the leaves.

### 2.2. Malondialdehyde and Carotenoid Content and Biomass Production in Carrots

**Figure 1:**
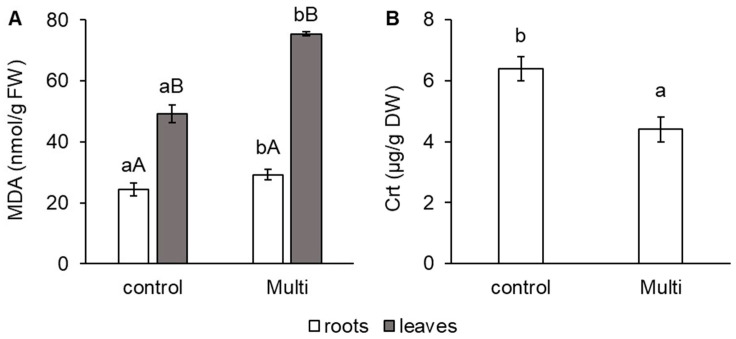
Content of (**A**) malondialdehyde (MDA, nmol/g FW) in carrot roots and leaves and (**B**) carotenoids (Crt, μg/g DW) in carrot roots from the control and multi-contaminated variant (Multi). The values represent the mean ± standard deviation (*n* = 4). Different letters indicate significant differences (*p* < 0.05) among variants (lowercase letters) and plant parts (uppercase letters).

To evaluate oxidative stress in carrots in relation to Cd, Pb and Zn accumulation, the change in malondialdehyde (MDA) content was determined and shown in Figure 1A. A significant increase in the MDA content of the Multi variant compared to the control was confirmed in the roots (by 20%), as well as in the leaves (by 53%). Compared with the roots, the leaves of both the control and the Multi variant showed a higher MDA content—by 102% and 158%, respectively (Figure 1A). Additionally, a change in carotenoids (Crt) was observed for the Multi variant in the carrot roots. These pigments decreased by 31% in the Multi variant compared with the control (Figure 1B).

**Figure 2:**
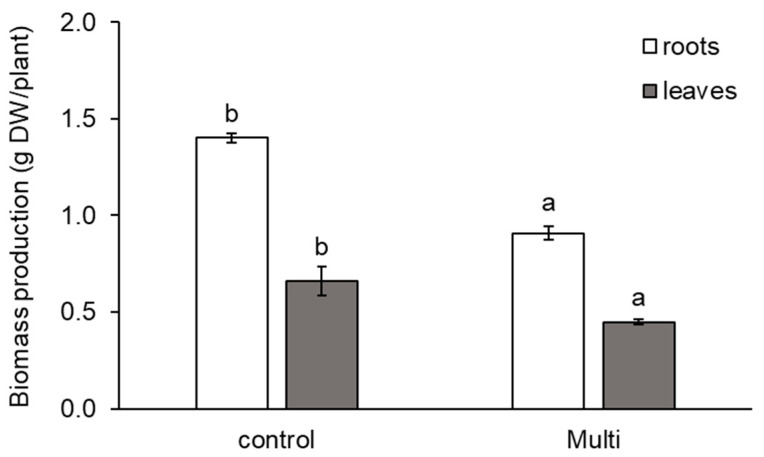
Production of dry weight (DW, g DW/plant) of carrot roots and leaves from the control and multi-contaminated variant (Multi). The values represent the mean ± standard deviation (*n* = 4). Different letters indicate significant differences (*p* < 0.05) among variants.

As shown in Figure 2, the accumulation of Cd, Pb and Zn also affected carrot biomass production. Our results showed a significant reduction in the dry weight (DW) of the roots and leaves by 35% and 32%, respectively.

### 2.3. Change in Gas-Exchange Parameters, Chlorophyll Fluorescence and Photosynthetic Pigments

The Multi variant significantly influenced the gas-exchange parameters (GAPs) of carrot leaves, except for the transpiration rate (*E*), as shown in Table 2. Compared with the control, a significant decrease in the Multi variant was observed for the intercellular CO_2_ concentration (*C*_i_; by 6%), stomatal conductance (*g*_s_; by 25%), leaf CO_2_ uptake rate (*P*_N_; by 11%) and chlorophyll fluorescence (F_v_/F_m_; by 12.5%).

A similar trend was observed regarding the effect of the Multi variant on the content of chlorophyll *a* (Chl *a*), as shown in Table 2. A significant decrease in Chl *a* of 28% was confirmed for the Multi variant compared with the control. In contrast, the content of chlorophyll *b* (Chl *b*) increased by 36% in the Multi variant compared with the control (Table 2). In relation to lower Chl *a* content due to accumulation of Cd, Pb and Zn, visible chlorosis was observed on the leaves of the Multi variant.

### 2.4. Change in Free Amino Acid Content

The accumulation of Cd, Pb and Zn influenced the free AA metabolism in carrot roots and leaves. Table 3 shows the effect of the Multi variant on the total content of free AAs, a group of transport AAs, specific free AAs and free AAs related to oxidative stress. The total content of free AAs was reduced in carrot roots and elevated in the leaves by the Multi variant, but the difference between variants was not statistically significant. A similar trend was observed for the group of transport AAs in carrot leaves. Compared with the control, the Multi variant reduced the content of transport AAs by 24% but not significantly. A decrease in the content of transport AAs by the Multi variant was observed in leaves (by 21%) compared with the control. These results indicate a higher sensitivity of carrot leaves to multi-contamination by Cd, Pb and Zn.

As shown in Table 3, the content of free AAs related to the adaptation of plants to stress was not significantly affected by the Multi variant in carrot roots, except for free proline (PRO). However, the majority of this group was increased by the Multi variant compared to the control in carrot leaves, except for free glycine (GLY) and free hydroxyproline (HYP) with no significant change. Different reactions to stress by multi-contamination between carrot roots and leaves were observed in the content of HYP and free sarcosine (SAR). These free AAs were below the detection limit in carrot roots, but they were determined in carrot leaves. In addition, SAR was measurable only in leaves of the Multi variant.

The PRO content was decreased by the Multi variant in carrot roots (by 46%), while in carrot leaves, it was significantly increased by 95% (Table 3). Similarly, free ornithine (ORN), which is related to PRO synthesis, was increased by the Multi variant compared with the control, but the 25% increase was significant only in carrot leaves. A similar trend was observed for the content of free methionine (MET)—a significant 31% increase confirmed only in leaves (Table 3).

### 2.5. Morphological and Anatomical Conditions of Carrot Roots and Changes in Enzyme Activities in the Root Zone

**Figure 3:**
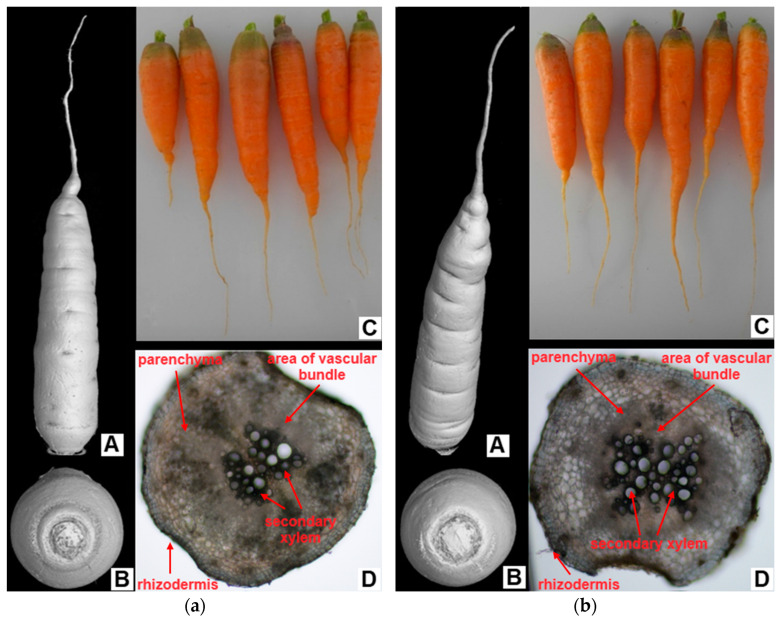
Morphological and anatomical structure of carrot roots from the (**a**) control and (**b**) multi-contaminated treatment—(**A**) 3D scan of carrots; (**B**) 3D scan of the top part of carrots; (**C**) photo of carrot after harvest; (**D**) cross-section through tap root (transversely, 5 mm from the tip of the root, 100× magnification).

No visible changes in the morphology of the carrot roots exposed to the Multi variant were observed (Figure 3A–C). In addition, in the anatomy, there were no significant differences between the variants in the observed structures (differentiation of xylem, parenchyma cortex, differentiation of emerging exodermis/corking of cell walls), as shown in Figure 3D.

**Figure 4:**
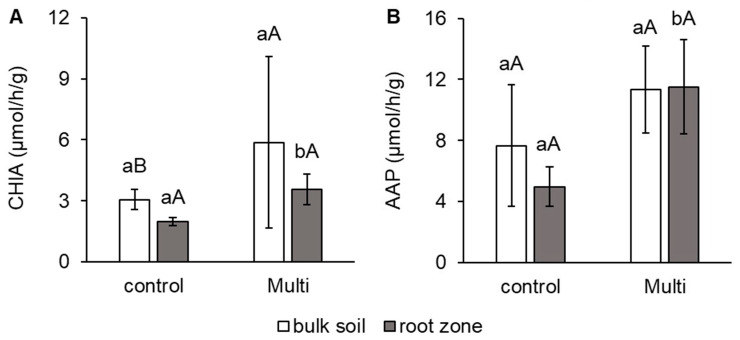
The activity of (**A**) chitinase (CHIA, μmol/h/g) and (**B**) alanine aminopeptidase (AAP, μmol/h/g) in bulk soil and the carrot root zone. The values represent the mean ± standard deviation (*n* = 4). Different letters indicate significant differences (*p* < 0.05) among variants (lowercase letters) and bulk soil/root zone (uppercase letters).

**Figure 5:**
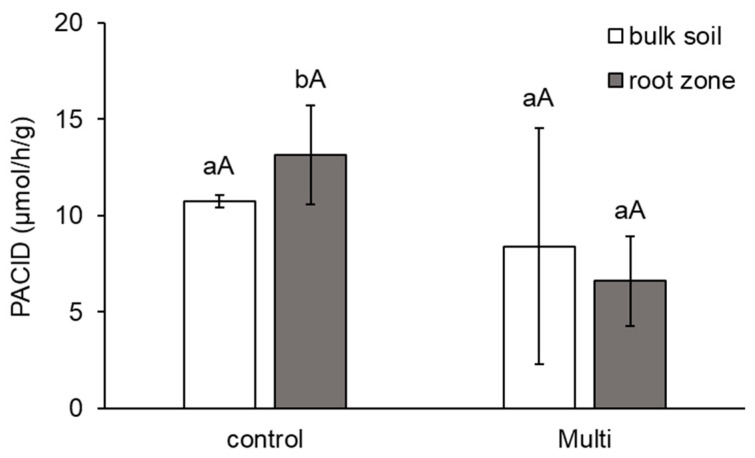
The activity of acid phosphatase (PACID, μmol/h/g)) in bulk soil and the carrot root zone. The values represent the mean ± standard deviation (*n* = 4). Different letters indicate significant differences (*p* < 0.05) among variants (lowercase letters) and bulk soil/root zone (uppercase letters).

Despite the fact that the morphological and anatomical condition of carrot roots was not significantly affected, changes in the root zone of carrots were determined. As shown in Figure 4 and Figure 5, the activity of some important soil enzymes was affected by the Multi variant, which contained significantly more Cd, Pb and Zn compared to the control soil. The results proved changes in enzymatic activities mainly in the root zone, in contrast to bulk soil. Significant differences in enzymatic activities between variants were confirmed only in the root zone. Unlike bulk soil, in the root zone, a significant increase in CHIA and AAP activities of 81% and 132%, respectively, was observed for the Multi variant compared to the control (Figure 4A,B). No significant effect between bulk soil and the root zone in enzyme activity was found in each variant.

As shown in Figure 5, PACID activity was reduced in the bulk soil (by 22%) and root zone (by 50%), but significantly decreased only in the root zone. Differences between the root zones and bulk soil of each variant were not significant.

## 3. Discussion

### 3.1. Accumulation and Translocation of Cd, Pb and Zn in Carrots

Our research confirmed that TE accumulation by plants largely depends on soil TE concentrations, which has been confirmed by other studies [44,45]. The accumulation of certain TEs by plants, such as Zn, is conditioned by the physiological needs of plants, but the uptake of necessary minerals by plants also causes the accumulation of unsatisfactory TEs from the soil [46].

Our results clearly demonstrated the highest accumulation of Cd and Zn in leaves compared to roots and periderm in the Multi variant. This high translocation from roots to leaves also confirmed the translocation factor. Bakhshayesh et al. [47] observed the effect of different Cd concentrations in soil samples on the corresponding level of Cd accumulation in selected vegetables (carrot, tarragon, basil, garlic, broccoli, spinach and dill). The highest Cd concentration was confirmed in the leaves, and the lowest Cd concentration was determined in the roots. However, Yang et al. [48] found that at concentrations of 3.0 and 9.0 mg Cd/kg soil, accumulation in roots was higher, while our Cd concentration was 6.5 mg/kg. In their research, translocation from roots to leaves occurred only at lower contamination levels (1.0 mg/kg). In the case of Zn accumulation and translocation, Roy and McDonald [43] observed that carrot, lettuce and spinach had higher Zn concentrations in leaves than in roots. The higher transfer and accumulation of Zn in leaves was probably because Zn is necessary for plant growth and development such as photosynthesis, ATP synthesis and various enzymatic reactions [24]. The availability of Cd and Zn for roots is also affected by their interaction, which is usually synergistic in nature due to their similar geochemistry [26]. According to Yang et al. [27], when the Cd content in soil was 200 times less than Zn, Zn limited Cd uptake by vegetables; Zn had no effect on Cd accumulation when the soil Cd/Zn ratio was close to 0.01, and Zn promoted Cd accumulation in vegetables when the Cd/Zn ratio was higher than 0.02. A ratio higher than 0.02 was calculated for the Multi variant (ratio 0.026) in our experiment, suggesting that Cd accumulation was promoted.

Carrot is a vegetable with a high ability to accumulate Pb in the roots. Our results are consistent with those published by Basu et al. [49]. The low mobility of Pb has been confirmed by other studies [15,43,50]. The uptake of Pb is based mainly on plant species and the interaction between roots (structures and great secretion of root exudates) and the rhizosphere (biochemical properties). Roots respond to the presence of Pb by forming a mechanical barrier. In some plants, including carrots, there is synthesis and deposition of callose (1,3-beta-glucan) between the plasma membrane and the cell wall. This modification of cell wall properties functions as a barrier against TEs [34] because callose limits the movement of these elements into the cytoplasm. However, the increase in callose content in response to stress generally limits primary root growth [51]. Lead transport from roots to the leaves was immobilised by the formation of Pb complexes with organic acids (carboxylic and amino acids), affecting Pb mobility in plant vessels [52]. Additionally, Pb availability in the roots is affected by the presence of other TEs. Cadmium and Zn have antagonistic effects against Pb [53]. Tran et al. [54] observed that the presence of Zn reduced Pb accumulation in the biomass of spinach, lettuce, potato and carrot, and this descent was higher with increasing soil Zn content.

In the leaves, Pb accumulation was significantly lower than in the roots. The limitation of Pb transport into leaves is significantly impaired by the existence of mechanical barriers, such as the endoderm (including Casparian strips) [55]. The formation of Pb phytate compounds in the xylem can affect its transport from the roots to the leaves [54]. 

### 3.2. Response of Malondialdehyde and Carotenoids to Cd, Pb and Zn and Biomass Production of Carrot

Malondialdehyde is a degradation product of lipid peroxidation induced by oxidative stress, and its content may reflect the degree of oxidative damage in plants. In addition, MDA is an indicator of free radical production and subsequent tissue damage [56,57]. An increase in the level of MDA under TE stress (e.g., Pb and Zn) has been reported in many plants [33,58,59,60]. An et al. [61] confirmed that MDA content increased significantly in carrots under high-concentration mixed contamination. The increase was lower than that in the treatments with the addition of one element. The authors speculated that certain concentrations of Cd and Cu share common uptake and transport sites in carrot cells and that they compete for cell binding, thereby reducing the toxicity with mixtures of Cd and Cu. This result might be explained by an antagonistic reaction between elements (Cd and Pb, Cd and Zn) [62] that alleviates the toxicity of the TEs to some extent. Our results confirmed a significant increase in MDA content in the roots and leaves of the Multi variant in contrast to the control (Figure 1A). An increase in the amount of ROS often leads to inhibited root growth [48], which, according to our results, was proved by the yield of dry biomass. However, other results indicate no significant impact on carrots from an anatomical or morphological point of view. Thus, the increased MDA content indicates the peroxidation of membrane lipids [63].

Carrot roots are characterised by a high quantity of Crt—coloured isoprenoid pigments. They act as colourants, precursors for plant isoprenoid volatiles and signalling molecules (abscisic acid and strigolactones), nutritional antioxidants and vitamin A precursors. Knowledge about the impact of environmental factors on their accumulation in carrot roots is limited. However, Crt plays multiple roles as an antioxidant molecule. These pigments are involved in ROS detoxification [64]. Our finding—the decrease in Crt by the Multi variant—is in accordance with Faiz et al. [65], who showed that Pb toxicity decreased the Crt content in carrot roots. The close relationship between Cd contamination and Crt in carrot roots was confirmed by Sharma et al. [66].

The phytotoxic effects of TEs on plant growth and development are commonly observed. However, this varies widely depending on the plant genotype, TE species, TE concentration and presence of other TEs [3,35,67,68]. In our study, a significant decrease in dry biomass was observed in the leaves, which could be caused by the high translocation of Cd, as well as in the roots (Figure 2). Similarly, Lima et al. [42] observed that carrots growing in Pb-contaminated soil had lower biomass production than in non-contaminated soil, but this decrease was not statistically confirmed. The decrease in carrot growth might be partly a consequence of Cd, Pb and Zn effects on photosynthesis, as well as interference in the biosynthesis of photosynthetic pigments.

### 3.3. Response of Gas-Exchange Parameters and Photosynthetic Pigments to Cd, Pb and Zn

Photosynthesis, the most important physiological process of plants, is highly sensitive to abiotic stress, such as TE toxicity [69]. Toxic elements interfere with plant photosynthesis by disrupting chloroplast ultrastructure, inhibiting light energy uptake, disrupting the electron transport chain and reducing the activity of enzymes associated with the Calvin cycle [23,28,29,70]. Our results demonstrated that TEs in soil have a significant impact on the gas-exchange parameters (GAPs) of carrots (Table 2). The *C*_i_ value was significantly higher in the Multi variant. Similar results were published for tobacco cultivated under Zn stress [19] and lettuce in a multi-contaminated hydroponic solution [71]. According to Yang et al. [72], the increase in *C*_i_ and decrease in *P*_N_ in *Pennisetum* sp. under Cd stress indicate that the plant is mainly affected by non-stomatal restriction. The increase in *C*_i_ in wheat cultivated under Cd stress indicates the immobilisation of CO_2_ in chloroplasts [73]. Bernardini et al. [74] observed a significant reduction in the GAP of *Phragmites australis* plants treated with Zn. They attributed this mainly to stomatal limitations due to Zn, as no increase in *C*_i_ was measured. Yang et al. [75] observed, in comparison to the control, a lower GAP of *Davidia involucrata* by individual Cd and Pb stress, while combined stress did not decrease the GAP to its minimum. According to these authors, the combination of Cd and Pb stress may have reduced the toxicity of TEs. In accordance with our results, He and Ren [76] confirmed decreasing *g*_s_ and *E* values in lettuce with increasing Cd content in the hydroponic solution. A decline in both mentioned parameters was also found in seedlings cultivated in soil contaminated with Pb, Zn and Cu [77]. Sharma et al. [66] showed that *E* decreased significantly in single and combined treatments of Cd and Zn. This decline could be the result of the TEs’ effect on water flow through the root and stomatal aperture.

Studies have shown that photosynthetic pigment content may reflect plant sensitivity to stress conditions, including TEs. Chl *a* and *b* are the main pigments of photosynthesis and are important indicators for characterising the physiological state of plant photosynthetic tissues [78]. The decline in photosynthetic pigments is most probably due to the inhibition of the reductive steps in the biosynthetic pathways of photosynthetic pigments due to the high redox potential of many TEs, such as Cd and Zn [79].

Chl *a* and *b* are highly susceptible to TE stress [80]. According to Huihui et al. [81], higher concentrations of Pb and Cd reduced chlorophyll content in mulberry seedling leaves, especially Chl *a,* which seemed to be more sensitive to TEs than Chl *b*. In our study, a significant decrease in Chl *a* and an increase in Chl *b* were observed in the Multi variant (Table 2). Similarly, Sharma et al. [66] observed a decrease in Chl *a* and *b* by individual Cd and Zn stress, as well as combined Cd and Zn stress, in carrot leaves. According to these authors, the decline in chlorophyll content may be related to the interaction of Cd or Zn with the –SH group of chlorophyll-synthesising enzymes during the various steps of chlorophyll biosynthesis. Additionally, Dahlawi et al. [82] observed a decrease in chlorophyll content in two genotypes of *Brassica campestris* caused by Cd, Pb and Ni toxicity, which also caused reduced photosynthetic activity. Alamer and Galal [80] found a significant decrease in Chl *a* and *b* content in eggplants caused by TE toxicity.

### 3.4. Response of Free Amino Acids to Cd, Pb and Zn

Amino acids play an important role in metal binding, antioxidant defence and signalling in plants under TE stress [83,84]. Therefore, plants exposed to TEs accumulate specific AAs, which may play an important role in their detoxification. Changes in free AA content in plants can be the result of regulation in their biosynthesis, an increase in AA synthesis or hydrolysis of proteins during plant stress responses [85]. In this study, a decline in the total content of free AAs was observed in the roots, but this was not a statistically significant difference between the variants (Table 3). Similarly, the decrease in free AA content was confirmed in edible parts of carrot, radish and lettuce growing on strong multi-contaminated soil [86]. Zhu et al. [87] observed a significant decrease in AAs in the roots of *Crassocephalum crepidioides* caused by Cd applied to soil. However, they observed an increasing trend in the leaves, except for the variant with 30 mg Cd/kg in the soil, where the total content of free AAs was significantly lower than in the control. A similar trend was observed in carrot leaves in our study, but the difference was not statistically significant.

Transport AAs are the AA group assimilated from ammonia into an organic form, which serves as the N donor in the biosynthesis of essentially all AAs and other nitrogen-containing compounds. Zemanová et al. [88] indicated a decrease in these AAs in *Arabidopsis halleri* and *Noccaea caerulescens* under Cd stress and confirmed the significant relationships between glutamic acid concentrations in both plants and biomass yield, as well as between glutamic acid concentrations and Cd content. The high Zn content in tobacco plants resulted in changes in the transport AAs [19]. In carrot roots, the content of the transport AA group showed the same trend as the total content of free AAs. In contrast to this trend, a decrease in this group was determined in leaves of the Multi variant, indicating a change in the regulation of N metabolism and a higher sensitivity of carrot leaves than roots to TEs in the soil. Additionally, the accumulation of other free AAs, such as PRO, ORN, GLY and MET, as well as specific free AAs—HYP and SAR—may reflect the higher sensitivity of carrot leaves to Cd, Pb and Zn in comparison to roots [89]. HYP, along with SAR, was below the detection limit in the roots. A detectable amount of HYP was observed in leaves with a decrease in the Multi variant, but this was not a significant difference. SAR was detected only in the leaves of the Multi variant. Pavlíková et al. [90] observed that SAR and HYP were not detected in the As hyperaccumulator *Pteris cretica* or the non-hyperaccumulator *P. straminea*. However, SAR content was confirmed in hyperaccumulators of *Noccaea* species [83], and the authors assumed that SAR could form complexes with Cd and other metals and protect nucleic acids from oxidative stress [91]. The authors also speculated that SAR formation is strongly dependent on the concentration of the radical species. The presence of specific AAs, such as SAR, occurring infrequently and/or at very low levels in plant stress metabolism, can be explained by epigenetic changes induced by stress in plants [84].

Polypeptides and proteins with a high content of PRO, GLY, MET and HYP play an important role in the growth of plant cell walls and adaptation to stress [83]. Proline is a multifunctional AA that acts as an osmoprotective molecule that protects plants from the harmful effects of ROS generated by abiotic stress, such as TEs. This was confirmed by Pavlíková et al. [19], who showed that the PRO content in the leaves of tobacco increased with an increasing Zn dose. Our results also demonstrated a significant increase in PRO in carrot leaves, while PRO content in roots decreased with Cd, Pb and Zn. Proline is synthesised in the cytosol from either L-glutamic acid or L-ornithine. Allosteric regulation of glutamate kinase activity by free PRO enables an increase in the content of glutamate that is required for the formation of a peptide bond between the g-carboxyl group of glutamate and the a-amino group of cysteine and is used in the synthesis of glutathione and phytochelatins in plant cells [92].

A significant difference was observed for ORN content in carrot leaves that increased with Cd, Pb and Zn in the soil (Table 3). A similar trend was observed in carrot roots, but the difference was not statistically significant. Ornithine is an alternative source of PRO biosynthesis [93]. The increased ORN content was determined 24 and 72 h after Zn treatment in pumpkin by Deng et al. [94].

In our study, AAs important for defence against TE stress—GLY and MET—showed a similar trend in response to Cd, Pb and Zn in carrot leaves, while the trend in the roots was the opposite (Table 3). Glycine is a crucial AA for the biosynthesis of cysteine and MET via serine [83]. Cysteine is a key precursor of phytochelatins, which bind Cd^2+^ in plant cells and thereby reduce its toxicity. In addition, GLY is an important exudate that reduces TE mobility in the root zone and bulk soil [95]. In the leaves, the content of GLY and MET was stimulated under Cd, Pb and Zn stress; however, the change was significant only for MET (Table 3). Zemanová et al. [96] observed changes in MET content in *Arabidopsis halleri* and *Thlaspi caerulescens*. MET was differentially regulated between the tested plant species, as its content was determined only in the roots of *A. halleri* after 30 and 60 days of plant cultivation. In this case, after 30 days, the MET content in variants with applied Cd increased, while after 60 days, a decreasing trend was demonstrated with increasing Cd content in the soil.

### 3.5. Morphological and Anatomical Conditions of Roots and Changes in Enzyme Activities under Cd, Pb and Zn Stress

The results of cross-sections and computed tomography (CT) visualisation demonstrated no visible difference between the carrots of both variants in terms of anatomy and morphology (Figure 3). However, during the anatomical analysis of *Tritonia* roots, Lux et al. [97] demonstrated reduced development of endodermal tissues in the contracting basal root parts, terminating their development with Casparian bands and weak deposits of suberin lamellae caused by Cd toxicity. Root length inhibition is one of the most frequently described symptoms caused by TEs, especially Cd [98,99]. Kováčik and Babula [100] observed a clear reduction in the growth of maize roots and shoots at the highest applied dose of Cd (100 μM). Bharwana et al. [60] confirmed Pb toxicity in cotton plants. As the dose of Pb increased, the length of the aboveground biomass and roots, as well as the leaf area and the number of leaves decreased compared to the control. In contrast to these results, the root length of carrots in the Multi variant was significantly higher.

Plant roots can significantly alter conditions in the root zone by releasing exudates, such as organic acids. This can stimulate microorganisms to produce soil enzymes [101,102]. Additionally, soil enzyme activities are potentially valuable indicators of soil health in cases of TE pollution. Our results showed significant changes in the enzymatic activity of CHIA, AAP and PACID in the root zone. Despite this, the difference between bulk soil and the root zone in CHIA, AAP and PACID activities was not statistically significant.

In our study, CHIA and AAP activities in the contaminated soil were significantly higher (Figure 4), while PACID was significantly lower (Figure 5). Wahsha et al. [103] observed that CHIA activity varied proportionally with TE (Ni, Cr, Cu, Pb, Zn, Fe, Mn) content in experimental soils and was negatively correlated. Controversially, Aponte et al. [104] concluded from their meta-analysis of collected data on enzymatic activities that enzymes involved in N (just like AAP and CHIA) and P (PACID) cycling in soil are not as significantly affected by TEs as those involved in C and S cycling. Ciadamidaro et al. [105] reported that protease activity (including AAP) was stimulated by the release of poplar root exudates. Moreover, when protein is broken down into oligopeptides or AAs by AAP, these simpler organic nitrogenous compounds can be directly absorbed and metabolised by soil microorganisms [7]. CHIA helps improve plant growth by improving soil conditions and soil quality by improving N availability to the plant and by breaking down chitin [106]. Chowdhury and Rasid [8] confirmed a decrease in PACID in agricultural soils contaminated with TEs. In addition to exudates, acid phosphatase is primarily produced by plants in the root zone [107].

It should be mentioned that soil enzymes are not only influenced by TEs but also by other factors, such as soil pH, organic matter content/quality, clay content, temperature, C and N content, salt type and content, specific vegetation, climatic conditions, and land management [106,108].

## 4. Materials and Methods

### 4.1. Soil Sampling and Characterisation

Multi-contaminated soil was collected from the Podlesí locality in the Příbram district (49°42′24″ N, 13 °58′32″ E) in the Czech Republic. This highly contaminated soil is influenced by the atmospheric deposition of TEs originating from historic lead–silver mining and smelting activities [109,110]. The main contaminants are Cd, Pb and Zn. Uncontaminated soil was collected from the Suchdol locality in the Prague district (50°8′8″ N, 14°22′43″ E) of the Czech Republic. This soil contained acceptable levels of TEs, as defined by Czech legislation [111]. In each locality, soil was collected from the top 30 cm, air-dried and homogenised. Basic soil characteristics and TE content were determined after sieving through a 2 mm mesh (Table 4). The pseudo-total content of TEs was determined by ICP–MS (Agilent 7700x, Agilent Technologies Inc., Santa Clara, CA, USA) after microwave-assisted aqua regia extraction [112]. The water-soluble fraction of TEs was extracted with demineralised water (1:5, *w/v*; 30 min shaking; 12 h equilibration; centrifugation at 5000 rpm) and determined in the supernatant using ICP-OES (Agilent 720; Agilent Technologies Inc., Santa Clara, CA, USA). The soil pH was measured in water extracts (1:5, *w/v*; 60 min shaking; 1 h equilibration) using a pH 8+ DHS metre (XS Instruments, Carpi, Italy). The total content of carbon was determined using a CHNS Vario MACRO cube analyser (Elemental Analyzer System GmbH, Hanau, Germany).

### 4.2. Experimental Design and Plant Material

A pot experiment was created with four independent replications of two variants: (i) control—uncontaminated soil and (ii) Multi—multiply contaminated soil (Cd, Pb and Zn). Each pot contained 5 kg of soil. The pots (6 L) were fertilised with 0.5 g N (in the form of NH_4_NO_3_), 0.16 g P and 0.4 g K (in the form of K_2_HPO_4_). Carrot (*Daucus carota* L., cv. NANTES 5) seeds were purchased from the SEMO a.s. company store (Smržice, Czech Republic) and sown directly into the soil in pots (15 seeds per pot). Thinning was performed after two true leaves developed, and six seedlings were maintained in each pot. Plants were grown under semi-controlled conditions in a greenhouse (natural photoperiod; day/night temperature 20–22 °C/15–18 °C; relative humidity ~60%) and irrigated with tap water, demineralised by reverse osmosis, until 50% of the soil water-holding capacity was reached (controlled gravimetrically every second day) [113]. Plants were harvested after 14 weeks of growth. Plants were divided into leaves and roots and weighed. The samples were partitioned for further analysis. One portion was immediately frozen in liquid nitrogen and stored at −80 °C until analysis of malondialdehyde and free amino acids, while the other portion was oven-dried at 40 °C to constant weight and homogenised for analysis of the elements, Crt, and chlorophylls.

### 4.3. Determination of Toxic Elements

The Cd, Pb and Zn content was determined according to Pavlíková et al. [86]. Briefly, homogenised dry material (0.5 ± 0.05 g) was digested in 10 mL of a mixture of HNO_3_ and H_2_O_2_ (4:1, *v/v*) in an Ethos 1 device (MLS GmbH, Leutkirch im Allgäu, Germany). The element content was determined using an Agilent 720 inductively coupled plasma optical emission spectrometer (ICP-OES; Agilent Technologies Inc., Santa Clara, CA, USA). Certified reference material (CRM NIST 1573a Tomato leaves and CRM NIST 1570a Spinach leaves, Analytika^®^, Prague, Czech Republic) was mineralised under the same conditions for quality assurance. 

The translocation factors of Cd, Pb and Zn were calculated by the following formula:

TF = C_leaves_/C_roots_, (1)

where C_leaves_ is the content of Cd, Pb and Zn determined in leaves and Cd_roots_ is the content of Cd, Pb and Zn determined in roots.

### 4.4. Determination of Malondialdehyde Content

The MDA content was measured according to Lhotská et al. [114]. In brief, fresh leaves (0.4 g) were homogenised with liquid nitrogen and 80% ethanol and centrifuged (2 mL microcentrifuge tubes, 5 min at 6000 rpm). Aliquots of 0.7 mL of each supernatant were mixed with 0.7 mL of 0.65% thiobarbituric acid in 20% trichloroacetic acid and 0.01% butylated hydroxytoluene, and a second set of 0.7 mL samples was mixed with 0.7 mL of 20% trichloroacetic acid and 0.01% butylated hydroxytoluene. The samples were incubated at 95 °C for 25 min, cooled, centrifuged (5 min at 6000 rpm) and measured on a UV-VIS spectrophotometer (Evolution 201, Thermo Fisher Scientific, Waltham, MA, USA) at 440, 532 and 600 nm.

### 4.5. Determination of Carotenoid and Chlorophyll Content

The Crt content in the roots and Chl *a* and *b* in the leaves were determined in dry biomass (0.5 ± 0.05 g) using a Soxhlet extractor. Samples were extracted with 80% acetone to complete extraction of all pigments from the biomass (60 min), diluted to 100 mL by the same extractant and measured on a UV-VIS spectrophotometer (Evolution 201, Thermo Fisher Scientific, Waltham, MA, USA). The Crt content was calculated according to Wellburn [115], and Chl *a* and *b* content was measured according to Porra et al. [116].

### 4.6. Determination of Gas-Exchange Parameters and Chlorophyll Fluorescence

The GAPs—*C*_i_ (μmol CO_2_/mol), *E* (mmol H_2_O/m^2^/s), *g*_s_ (mol H_2_O/m^2^/s) and *P*_N_ (μmol/CO_2_/m^2^/s)—were determined in situ using the portable gas-exchange system LCpro+ (ADC BioScientific, Ltd., Hoddesdon, UK). The duration of each individual measurement was 10 min after the establishment of steady-state conditions inside the measurement chamber. The conditions in the chamber were 25 °C, ambient CO_2_ concentration (550 ± 50 μL/L), airflow rate of 205 ± 30 μmol/s and photosynthetically active radiation of 650 ± 50/μmol/m^2^/s. All measurements were conducted between 08:00 and 11:30 Central European Time (CET).

Chlorophyll fluorescence was measured in situ using a portable fluorometer (OS1-FL; Opti-Sciences, ADC, BioScientific, Ltd., Hoddesdon, UK). The leaves were shaded for 20 min using clips to set up a dark-adapted state. Chlorophyll fluorescence was excited using a 660 nm solid-state light source, with filters blocking radiation longer than 690 nm. The saturation of the measured photosystem was achieved using a filtered 35 W halogen lamp (350–690 nm) with a pulse of 15,000 μmol/m^2^/s for 0.8 s. Subsequently, F_v_/F_m_ was calculated using the following equation: F_v_/F_m_ = (F_m_ − F_0_)/F_m_.

### 4.7. Determination of Free Amino Acids

The free AA content in carrot leaves and roots was determined according to Pavlíková et al. [113]. In brief, after extraction, the samples were derivatised by an EZ:faast kit (Phenomenex, Torrance, CA, USA) and analysed using a Hewlett Packard 6890N/5975 MSD gas chromatography–mass spectrometry system (GC-MS; Agilent Technologies Inc., Santa Clara, CA, USA) with a ZB-AAA 10 m × 0.25 mm AA analysis GC column.

### 4.8. Evaluation of Morphology and Anatomy of Carrot Roots

The morphology of the carrot roots was evaluated using an X-ray CT. The X-ray system used was the industrial X-CT system XT H 225ST (Nikon Metrology, Leuven, Belgium) with a power source of 59.9 W at 180 kV and 333 μA. The sample was rotated over 360° by angular steps of 0.25°. As such, 1441 images were generated for one tomogram dataset. Each image has 2850 × 2850 pixels with a spatial resolution of approximately 43.34 μm. One tomographic scan lasted for 30 min. Afterwards, all 2D pictures were reconstructed into a 3D model (CT Pro 3D 5.3.2, Nikon Metrology), and the model was presented and analysed using voxel analysis software (VGStudio MAX 2023.1, Volume Graphics GmbH, Heidelberg, Germany).

The anatomy of carrot roots was evaluated using microscopic cross-sections. Samples were obtained 5 mm from the tip of the tap root and observed without staining at 100× magnification using a Nikon Eclipse 50i microscope with a Nikon DS-Fi2 camera (Nikon Corporation, Tokyo, Japan).

### 4.9. Determination of Soil Enzyme Activity

Samples of the root zone (defined as soil adhering to the roots after shaking) and bulk soil (soil outside the root zone) were collected from pots during plant harvesting. Before analysis, the soil was sieved (<2 mm), homogenised, lyophilised and stored at −80 °C.

The activities of CHIA, AAP and PACID in the root zone and bulk soil were determined according to Hanč and Hřebečková [117] with several modifications. The lyophilised soil (0.2 ± 0.002 g) was mixed with 20 mL phosphate buffer (c = 50 mmol/L, pH = 7.0). The mixture was homogenised using Ultra-Turrax (IKA Labortechnik, Staufen im Breisgau, Germany) for 30 s at 8000 rpm. Before measuring CHIA and AAP, homogenised samples were stored in a refrigerator for 24 h, while for PACID, they were stored for 2 h. To determine the activity of the individual soil enzymes, 10 mL of dimethyl sulphoxide and the substrate were prepared in a solution. The substrates used for individual soil enzymes were as follows: CHIA—3.79 mg 4-methylumbellyferyl-N-acetylglucosaminide (c = 1.00 mmol/L); AAP—9.01 mg L-alanine-7-amido-4-methylcoumarin (c = 2.50 mmol/L); PACID—7.04 mg 4-methylumbelliferyl-phosphate (c = 2.75 mmol/L). The homogenised suspension (200 Μl) and substrate solution (40 Μl) were pipetted into the relevant wells in the microplates. For PACID, an additional 20 Μl of dimethyl sulphoxide was pipetted. The microplate was incubated at 40 °C for 5 min (initial) and 120 min (final) in a Robbins Scientific^®^ 2000 micro hybridisation incubator (SciGene, Sunnyvale, CA, USA). Fluorescence was measured after each incubation using the tecan Infinite^®^ M200 (Tecan Austria GmbH, Salzburg, Austria). Enzymatic activity was calculated from the difference between the initial and final values (μmol/h/g) [118].

### 4.10. Statistical Analysis

Statistical processing of the results was performed using the Statistica 12.0 programme (StatSoft, Tulsa, OK, USA). Data are presented as the mean ± standard deviation (*n* = 4). A one-way analysis of variance (ANOVA) followed by a post hoc comparison Fisher’s LSD test (*p* < 0.05) was used to identify statistically significant differences.

## 5. Conclusions

The soils used for this experiment differed significantly due to TE contamination. The soil of the Multi variant was heavily polluted, and Cd, Zn and Pb content was significantly higher in contrast to acceptable levels of these elements in soil according to Czech legislation. Toxic element stress due to the accumulation of Tes alters physiological and metabolic processes in plants. Our results clearly demonstrated the highest accumulation of Cd and Zn in leaves compared to roots and the opposite trend for Pb accumulation. The presented data showed that mixed Cd, Zn and Pb exposure led to oxidative stress in carrots. The oxidative stress was confirmed by a significant accumulation of MDA and a decrease in carotenoid content in the roots of carrots growing under high-concentration mixed contamination. The Multi variant significantly influenced the GAP of carrot leaves and chlorophyll content, reflecting plant sensitivity to stress conditions. The TE stress led to a reduction in the total content of free Aas in carrot roots and to its elevation in the leaves by the Multi variant. The accumulation of free Aas, such as PRO, ORN, GLY and MET, as well as specific free Aas—HYP and SAR—may reflect the higher sensitivity of carrot leaves to Cd, Pb and Zn in comparison to roots. The morphological and anatomical condition of carrot roots was not significantly affected. The carrot root zone was also affected by high multi-contamination, which was shown by the change in important enzyme activities.

## Figures and Tables

**Table 1:** Toxic element content (mg/kg DW) in carrot roots, periderm and leaves from the control and multi-contaminated variant (Multi) and translocation factor (TF). The values represent the mean ± standard deviation (*n* = 4). Different letters in a row indicate significant differences (*p* < 0.05) among variants (lowercase letters) and plant parts (uppercase letters).

	Roots		Periderm		Leaves		TF	
	Control	Multi	Control	Multi	Control	Multi	Control	Multi
Cd (mg/kg DW)	0.2 ± 0.01 ^aA^	4.8 ± 0.5 ^bA^	0.2 ± 0.04 ^aAB^	8.4 ± 0.1 ^bB^	0.3 ± 0.1 ^aB^	13.8 ± 1.1 ^bC^	1.5	2.9
Pb (mg/kg DW)	<2.0	68.8 ± 1.6 ^C^	<2.0	16.3 ± 1.0 ^A^	<2.0	28.0 ± 1.5 ^B^	-	0.4
Zn (mg/kg DW)	10.3 ± 0.5 ^aA^	25.1 ± 1.0 ^bA^	17.2 ± 0.6 ^aB^	24.4 ± 0.6 ^bA^	16.9 ± 1.4 ^aB^	64.1 ± 2.3 ^bB^	1.6	2.6

**Table 2:** Intercellular CO_2_ concentration (*C*_i_), transpiration rate (*E*), stomatal conductance (*g*_s_), leaf CO_2_ uptake rate (*P*_N_), chlorophyll fluorescence (F_v_/F_m_), chlorophyll *a* content (Chl *a*) and chlorophyll *b* content (Chl *b*) in carrot leaves from the control and multi-contaminated variant (Multi). The values represent the mean ± standard deviation (*n* = 4). Letters in a row indicate significant differences (*p* < 0.05) among variants.

	Control	Multi
*C*_i_ (µmol CO_2_/mol)	309.3 ± 4.8 ^a^	328.8 ± 1.9 ^b^
*E* (mmol H_2_O/m^2^/s)	2.9 ± 0.04 ^a^	2.9 ± 0.02 ^a^
*g*_s_ (mol H_2_O/m^2^/s)	0.4 ± 0.03 ^b^	0.3 ± 0.01 ^a^
*P*_N_ (µmol CO_2_/m^2^/s)	12.5 ± 0.2 ^b^	11.1 ± 0.2 ^a^
F_v_/F_m_ (-)	0.8 ± 0.01 ^b^	0.7 ± 0.01 ^a^
Chl *a* (mg/kg DW)	1.8 ± 0.03 ^b^	1.6 ± 0.06 ^a^
Chl *b* (mg/kg DW)	0.9 ± 0.04 ^a^	1.1 ± 0.02 ^b^

**Table 3:** Content of free amino acids (AAs, μmol/kg FW) in carrot roots and leaves from the control and multi-contaminated variant (Multi)—total content of amino acids (Ʃ AAs), transport amino acids (transport AAs), proline (PRO), ornithine (ORN), glycine (GLY), methionine (MET), hydroxyproline (HYP) and sarcosine (SAR). The values represent the mean ± standard deviation (*n* = 4). Different letters indicate significant differences (*p* < 0.05) among variants. nd—value under the limit of detection.

		Control	Multi
Ʃ AAs (μmol/kg FW)	roots	30,600.6 ± 6996.8 ^a^	23,251.9 ± 2097.9 ^a^
	leaves	9871.7 ± 275.5 ^a^	10,327.5 ± 720.2 ^a^
transport AAs (μmol/kg FW)	roots	25,547.1 ± 6293.9 ^a^	19,305.3 ± 1411.6 ^a^
	leaves	7136.1 ± 252.8 ^b^	5661.5 ± 491.2 ^a^
PRO (μmol/kg FW)	roots	832.8 ± 61.7 ^b^	449.8 ± 235.6 ^a^
	leaves	82.5 ± 0.8 ^a^	161.3 ± 17.9 ^b^
ORN (μmol/kg FW)	roots	57.1 ± 8.6 ^a^	62.8 ± 7.9 ^a^
	leaves	56.9 ± 0.2 ^a^	71.4 ± 1.1 ^b^
GLY (μmol/kg FW)	roots	86.8 ± 4.5 ^a^	83.9 ± 6.6 ^a^
	leaves	161.7 ± 0.9 ^a^	182.0 ± 15.7 ^a^
MET (μmol/kg FW)	roots	71.7 ± 8.9 ^a^	77.7 ± 3.6 ^a^
	leaves	49.0 ± 0.3 ^a^	64.1 ± 0.2 ^b^
HYP (μmol/kg FW)	roots	nd	nd
	leaves	25.5 ± 1.8 ^a^	27.2 ± 3.5 ^a^
SAR (μmol/kg FW)	roots	nd	nd
	leaves	nd	73.7 ± 0.4

**Table 4:** Basic characteristics of experimental soils and toxic element content.

Parameters	Control	Multi
Soil type and subtype	Chernozem Haplic	Cambisol Haplic
pH_H2O_	7.5	6.0
Cation-Exchange Capacity (mmol_(+)_/kg)	230.1 ± 5.0	165.8 ± 15.1
Total Carbon (%)	2.0 ± 0.08	2.4 ± 0.04
Cd_pseudo-total_/Cd_water-soluble_ (mg/kg)	0.4 ± 0.01/<0.005	6.5 ± 1.1/0.01 ± 0.002
Pb_pseudo-total_/Pb_water-soluble_ (mg/kg)	36.6 ± 1.6/<0.1	1560.2 ± 167.1/0.4 ± 0.09
Zn_pseudo-total_/Zn_water-soluble_ (mg/kg)	93.1 ± 1.5/0.03 ± 0.01	243.4 ± 8.2/0.1 ± 0.03

Czech legislation limits for pseudo-total contents of elements are 0.4 and 0.5 mg/kg for Cd, 55 and 60 mg/kg for Pb, and 105 and 120 mg/kg for Zn in light-textured and other soils, respectively [111].

## Data Availability

The data presented in this study are available in the article.
